# Strangulated Hernia Reduced by Application of Cold Water

**Published:** 1871-04

**Authors:** 


Strangulated Hernia Reduced by Application of Cold Water.—Dr. P. Foster reports (*Lancet*, Aug. 27, 1870) two cases of this. In both cases the patients were advanced in life, (about sixty) and the hernia had existed in a reducible form for several years. After trying the taxis patiently for about half an hour in each case without producing the slightest effect, I applied cold water. In Mr. W——'s case the tumour disappeared in about ten minutes under the influence of cold water alone; in Mr. S——'s case, after about the same length of time, the reduction was effected almost immediately upon removing the cold water cloth and reapplying the taxis. The patients were placed in the position usually recommended, and the cold water was applied by means of a pocket-handkerchief, the application being renewed every two or three minutes.—*Ibid.*

